# Supplementary material for: Meta-Analyses of the Relationships between Family Systems Practices, Parents’ Psychological Health, and Parenting Quality
Source: Int J Environ Res Public Health. 2023 Sep 7;20(18):6723. doi: 10.3390/ijerph20186723 (PMC10530758; doi:10.3390/ijerph20186723)
Supplement: Supplementary file 1 [file ijerph-20-06723-s001.zip › ijerph-2310594-supplementary.pdf]

# Meta-Analyses of the Relationships Between Family Systems Practices, Parents' Psychological Health, and Parenting Quality: Supplemental Report

Carl J. Dunst
Senior Research Scientist
Orelena Hawks Puckett Institute
Asheville, North Carolina, USA
cdunst@puckett.org

This supplemental report includes additional information about the methodology and sources of data for the *Meta-Analyses of the Relationships Between Family Systems Practices, Parents' Psychological Health, and Parenting Quality* (Dunst, 2023). The supplemental report includes a brief description of the applied family systems theory that guided the conduct of the meta-analyses (Dunst, 2017, 2022b), a description of how family systems practices are hypothesized to be related to parents' psychological health and parenting quality, the foundations for how parents' psychological health is expected to be related to parenting quality, and the search strategy and results for identifying studies for investigating the relationships between parents' psychological health and parenting quality in households with children with identified disabilities, medical conditions, or at-risk for poor outcome conditions. Examples of psychological health and parenting quality measures that were used in the psychological health-parenting quality studies are also reported. The studies in the final meta-analysis of the relationships between different types of parents' psychological health (general health, depression, stress, well-being, caregiving burden, and parenting stress) and different types of parenting quality) parenting beliefs, parent involvement in children's learning and education, and parenting behavior and practices) are included in the supplemental report.

## Applied Family Systems Intervention Model

Figure 1 shows the family systems intervention model that has been the focus of research and practice by the author and his colleagues. The model includes four components: Family needs (concerns and priorities), the resources and social supports for needs satisfaction, family and family member strengths used to obtain resources and supports, and practitioner use of family-centered practices to build and strengthen family capacity to use strengths to procure needed resources and supports (see e.g., Dunst, 2000, 2017, 2022b; Dunst et al., 1988, 1994).

Different family and social systems models were used to develop the family systems intervention model where each of the components is hypothesized to be related to improved parent, family, parent-child, and child behavior, development, and well-being (e.g., Broderick, 1993; Bronfenbrenner, 1979; Johnson & Ray, 2016; Kerig, 2019). Research syntheses of studies of each of the intervention components indicate that a large number of unmet family needs are associated with poor functioning and that the presence and use of the other three practices are associated with positive functioning (see Dunst, 2023, for results from these syntheses). Results from structural equation modeling (Dunst, 2020; Dunst et al., 2007) and meta-analytic structural equation modeling (Dunst et al., 2019; Dunst & Trivette, 2009; Trivette et al., 2010) studies

show that the four family systems model practices are both directly and indirectly related to parent, family, and child functioning mediated by parents' belief appraisals and parents' psychological health.

Findings reported in Dunst (2023) extend this research by examining the relationships between parents' psychological health and parenting quality. The study also includes analyses to determine if parents' psychological health mediates the relationships between the core family systems intervention practices (needs, supports, resources, and strengths) and parenting quality. This supplemental report includes the procedures for identifying relevant studies, data coding and analysis, and the raw data that was used to conduct analyses of the relationships between different dimensions of parents' psychological health and parenting quality. The meta-analysis was conducted to address the "lack of understanding regarding the [relationships] between specific dimensions of parental mental health and parenting" and mechanisms for understanding how family systems intervention variables were directly and indirectly related to parenting quality as described in the call for papers for the special issue of IJERPH on ***Parenting and Mental Health***.

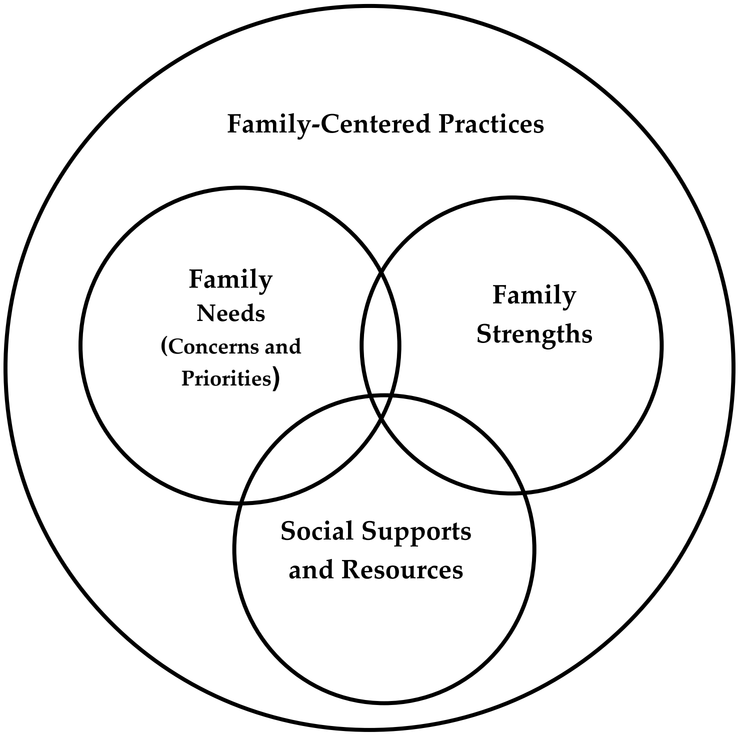


**Figure 1**. Major components of the applied family systems intervention practices model.

## Sources of Data

The data for the relationships between parents' psychological health and parenting quality came from two sources: (a) meta-analyses of studies of the relationships between family needs (Dunst, 2022d), family social support (Dunst, 2022a, 2022c, 2022e), family resources

(Dunst, 2021b, 2021c, 2022f), family strengths (Dunst, 2021a, 2021d; Dunst et al., 2021), and either or both parents' psychological health and parenting quality and (b) a meta-analysis of the relationships between parents' psychological health and parenting quality (Dunst, 2023). The search strategies and results for each of the family systems meta-analyses are included in the published reports. This supplemental report includes information for understanding the conduct of the meta-analyses of the relationships between parents' psychological health and parenting quality.

## Search Strategy

Four primary and four secondary search sources were used to locate relevant studies. The four primary sources were PsycNET, ProQuest Central, PubMed, and Educational Resource Information Center (ERIC). The secondary search sources were Google Scholar, Bielefeld Academic Search Engine (BASE), COnecting REpositories (CORE) search engine, and the Directory of Open Access Journals (DOAJ) search engine. Controlled vocabulary searches were used in the four primary search sources and natural language searches were used in both the primary and secondary search sources.

All searches included both psychological health terms and parenting quality terms. The psychological health terms included but were not limited to *psychological health, depression, stress, stressful life events, distress, anxiety, well-being, quality of life, positive affect, negative affect, parenting stress, parenting distress, caregiver burden, caregiving burden,* and *caregiving strain.* Searches were also conducted using the names of the psychological health measures in the different family systems meta-analyses cited above. The parenting quality terms included but were not limited to *parenting quality, parenting beliefs, parenting self-efficacy, parenting competence, parenting involvement, parental involvement, parenting engagement, parent-child engagement, parent-child activities, child routines, family routines, parenting styles, parent-child interactions, parenting responsiveness,* and *parenting sensitivity.* Searches also included the names of the parenting scales in the family systems meta-analyses.

In those cases where searches in any one database for the combination of psychological health and parenting quality terms resulted in more than 2000 results, the terms *developmental disabilities, identified disabilities, special needs children,special health care needs, medical conditions, chronic health conditions,* and *at-risk children* were added to delimit the search results.

## Search Results

Figure 2 shows the PRIMSA flow chart for the results from the literature searches. More than half of the nonduplicte records were excluded for not meeting inclusion criteria (see Dunst 2023). The records excluded during the screening phase were primarily because the report did not include a study of the relevant variables. The records excluded when full-text articles were assessed for eligibility were for the reasons listed in Figure 2. For example, studies were excluded when all of the sizes of effect between the psychological health and parenting quality measures were outliers. Studies that included both outliers and nonoutliers were included but the

nonoutliers for specific psychological health-parenting quality relationships were retained for further analysis.

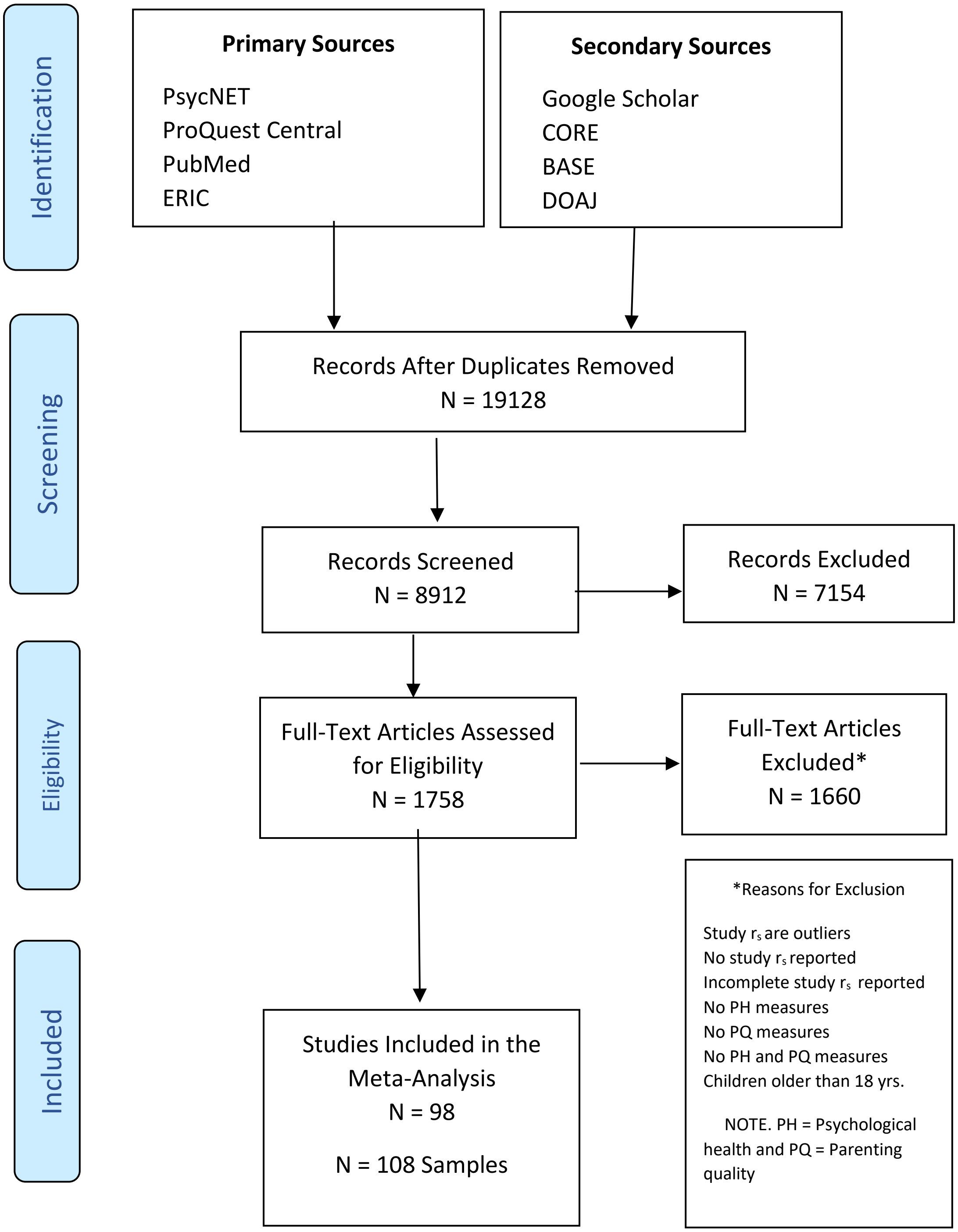


**Figure 2.** Flow chart for the identification of parenting quality studies. (Adapted from Moher et., 2009).

Ninety-eight studies met the inclusion criteria which are listed in Table 1. There were 108 independent samples of study participants. These samples were considered the number of studies for the meta-analysis of the relationships between parents' psychological health and parenting quality.

Table 1 shows selected characteristics of the studies. The table includes the country where the studies were conducted, the type of research report (peer-reviewed vs. non-peer-reviewed), the primary study participants and the percentage of the samples, the children's conditions (at-risk for poor outcomes, Autism Spectrum Disorders, behavior problems, developmental disabilities, medical conditions), and the ages of the children of the study participants.

Table 2 includes examples of the parents' psychological health measures and parenting quality measures that were the focus of analysis. The six health dimensions were general psychological health, depression, personal stress, well-being, parenting stress, and caregiving burden. The targets of appraisals of the latter two dimensions were child-related whereas the other dimensions were nonchild-specific. The three parenting quality measures were parenting beliefs, parental engagement, and parenting behavior and practices.

All of the psychological health measures, and both the parenting belief and parent involvement measures were completed by the study participants. The parenting behavior and practices measures include both self-report scales and investigator-administered observational scales of parenting behavior and practices.

## Methods of Analysis

*Meta-Essentials* was used to perform the meta-analyses of the relationships between the different dimensions of parents' psychological health and the different dimensions of parenting quality (Suurmond et al., 2017; Van Rhee et al., 2015). Separate analyses were conducted for the relationships between each psychological health measure and each parenting quality measure. Preliminary analyses were conducted to identify and remove outliers and influential cases (Viechtbauer & Cheung, 2010). This was done in a stepwise manner where the most extreme cases were removed first, the analyses rerun, and the next most extreme cases removed. This procedure was used until all outliers and influential cases were deleted.

The *Meta-Essentials* Excel Spreadsheets for the relationships between the six psychological health measures and the parenting beliefs, involvement, and parenting behavior and practices measures are included in Tables 3, 4, and 5 respectively. The input for the different pairwise combinations was the correlation coefficient and sample size reported in each study. The methods of analysis for the main effects and moderating effects are described in Dunst (2023).

**Table 1**
Selected Characteristics of the Studies of the Relationships Between Parents' Psychological Health and Parenting Quality

| Study | Country | Type of Report[a] | Primary Sample[b] | % of Sample | Child Condition[c] | Child Age (Years) |
|---|---|---|---|---|---|---|
| Almand (2004) | USA | MT | Mothers | 100 | DD | 2-6 |
| Anderson (2015) | USA | JA | Mothers | 94 | AR | 4-6 |
| Arnold et al. (2008) | USA | JA | Mothers | 96 | AR | 3-5 |
| Baker and Iruka (2013) | USA | JA | Mothers | 100 | AR | 4-6 |
| Bax (2005) Sample 1 | Canada | DD | Mothers | 100 | LR | 2-5 |
| Bax (2005) Sample 2 | Canada | DD | Fathers | 100 | LR | 2-5 |
| Benson (2015) | USA | JA | Mothers | 100 | ASD | 7-14 |
| Biondic (2019) | Canada | DD | Mothers | 57 | DD | 13-18 |
| Bishop (2016) | USA | MT | Mothers | 91 | MC | 1-3 |
| Bradley et al. (2023) | USA | JA | Mothers | 91 | ASD | <1-18 |
| Brody and Flor (1997) | USA | JA | Mothers | 100 | AR | 6-9 |
| Brody et al. (2006) | USA | JA | Mothers | 100 | AR | 11 |
| Cantwell et al. (2014) | Ireland | JA | Mothers | 91 | DD | <1-18 |
| Cardenas (2020) | USA | DD | Mothers | 100 | LR | <1-6 |
| Carreras et al. (2019) | USA | JA | Mothers | 92 | AR | 3-5 |
| Cejas et al. (2021) | USA | JA | Mothers | 100 | HL | <1-5 |
| Cekic and Karageyik (2021) | Turkey | JA | Mothers | 59 | AR | 8-16 |
| Chairinkam et al. (2021) | Thailand | JA | Mothers | 100 | MC | 1-6 |
| Chen et al. (2022) | China | JA | Mothers | 100 | ASD | 2-15 |
| Chiel (2018) | USA | DD | Mothers | 100 | ASD | 2-6 |
| Chisholm et al. (2014) | UK | JA | Mothers | 100 | MC | 4-8 |
| Collins-Allen (2006) | USA | DD | Mothers | 100 | DD | 2-5 |
| Comfort (1987) Sample 1 | USA | CP | Mothers | 100 | DD/AR | <1-3 |
| Comfort (1987) Sample 2 | USA | CP | Fathers | 100 | DD/AR | <1-3 |
| Cooke (2010) | USA | DD | Mothers | 84 | DD | 2-4 |
| Dahl (1993) | USA | DD | Mothers | 100 | BP | 3-8 |
| Dinehart et al. (2006) | USA | JA | Mothers | 55 | MC | 1-3 |
| Dissanayake et al. (2020) | Australia | JA | Mothers | 84 | ASD | 6-16 |
| Dubriwny and Hellman (2010) | USA | RR | NR | --- | AR | NR |
| Dunst (1985) Study 3 | USA | JA | Mothers | 94 | DD/AR | <1-7 |
| Dunst (1985) Study 4 | USA | JA | Mothers | 79 | DD | <1-5 |
| Dunst (1985) Study 5 | USA | JA | Mothers | 74 | DD | <1-5 |
| Dunst (1985) Study 6 | USA | JA | Mothers | 85 | DD | 2-17 |
| Dunst and Trivette (1988) | USA | BC | Mothers | 82 | DD | <1-5 |
| Ehrlick (2004) | USA | DD | Mothers | 63 | BP | 4-8 |
| Ellis (2019) | UK | DD | Mothers | 89 | DD | 4-17 |
| Emmen et al. (2013) | Netherlands | JA | Mothers | 100 | AR | 5-6 |
| Equihua (2010) | USA | MT | Mothers | 100 | AR | 6-10 |
| Fenning et al. (2014) Sample 1 | USA | JA | Mothers | 100 | DD | 5 |
| Fenning et al. (2014) Sample 2 | USA | JA | Fathers | 100 | DD | 5 |
| Fiese et al. (2008) | USA | JA | Mothers | 93 | MC | 5-13 |
| Foster et al. (2010) | USA | JA | Mothers | 87 | MC | 3-18 |
| Frank et al. (2017) | USA | JA | Mothers | 88 | BP | 6-18 |
| Gao et al. (2012) | China | JA | Mothers | 100 | LR | NA |
| Garcia-Lopez et al. (2016) Sample 1 | Spain | JA | Mothers | 100 | ASD | 3-17 |
| Garcia-Lopez et al. (2016) Sample 2 | Spain | JA | Fathers | 100 | ASD | 3-17 |
| Garland et al. (2013) | USA | JA | Mothers | 90 | BP | 3-5 |
| Gartstein and Sheeber (2004) | USA | JA | Mothers | 100 | BP | 3-6 |

**Table 1**, continued.

| Study | Country | Type of Report | Primary Sample | % of Sample | Child Condition | Child Age (Years) |
|---|---|---|---|---|---|---|
| Gavidia-Payne and Stoneman (1997) Sample 1 | USA | JA | Mothers | 100 | DD | <1-5 |
| Gavidia-Payne and Stoneman (1997) Sample 2 | USA | JA | Fathers | 100 | DD | <1-5 |
| Glenn et al. (2008) | UK | JA | Mothers | 100 | DD | 1-5 |
| Huang et al. (2017) | Uganda | JA | Mothers | 82 | AR | 3-8 |
| Huang et al. (2018) | Ghana | JA | Mothers | 64 | AR | 3-8 |
| Jandric and Kurtovic (2021) | Croatia | JA | Mothers | 74 | DD | 7-14 |
| Jordan (2003) | USA | DD | Mothers | 100 | BP | 6-12 |
| Karlioglu and Sari (2019) | Turkey | JA | Fathers | 100 | DD | <1-21 |
| Kelly et al. (2011) | USA | JA | GM | 100 | BP | 2-16 |
| Kilmer et al. (2010) | USA | JA | NR | NR | DD | 4-17 |
| Kim (2007) | Korea | DD | Mothers | 100 | AR | 3-5 |
| Kohl et al. (2000) | USA | JA | Mothers | 97 | AR | 5-6 |
| Lee et al. (2009) | USA | JA | Mothers | 92 | AR | 4-10 |
| Letiecq and Koblinsky (2003) | USA | JA | Fathers | 100 | AR | 3-5 |
| Lindsey and Barry (2018) | USA | JA | Mothers | 75 | ASD | 4-11 |
| Mahmoud et al. (2022) | Egypt | JA | Mothers | 73 | MC | <1-18 |
| McRae et al. (2020) | USA | JA | Mothers | 74 | DD | 6-12 |
| Merson (2012) | USA | DD | Mothers | 100 | DD | 5-13 |
| Mitchell (2011) | Australia | RR | Mothers | 91 | MC | 2-12 |
| Nievar et al. (2008) | USA | CP | Mothers | 100 | AR | 3-6 |
| Okado et al. (2014) | USA | JA | Mothers | 96 | AR | 4-6 |
| Palermo et al. (2017) | USA | JA | Mothers | 100 | AR | <1-3 |
| Piehler et al. (2014) | USA | JA | Mothers | 100 | BP | 5-8 |
| Platt et al. (2014) Sample 1 | USA | JA | Mothers | 100 | DD | NR |
| Platt er al. (2014) Sample 2 | USA | JA | Fathers | 100 | DD | NR |
| Podjarny (2007) | Canada | MT | Mothers | 91 | ASD | 3-6 |
| Quinn (2017) | Australia | HT | Mothers | 91 | ASD | NR |
| Raisanen (2013) | USA | MT | Mothers | 100 | AR | 4-17 |
| Richter et al. (2018) | Germany | JA | Mothers | 100 | LR | 5-7 |
| Robinson (2019) | Canada | DD | Mothers | 96 | ASD | 4-18 |
| Robokos (2012) | USA | BK | Mothers | 99 | AR | 0-1 |
| Rodenburg et al. (2007) | Netherlands | JA | Mothers | 89 | MC | 4-16 |
| Rogers et al. (2009) | Canada | JA | Mothers | 91 | DD | 8-12 |
| Rudelli et al. (2021) | Switzerland | JA | Fathers | 100 | ASD | 3-16 |
| Sajedi et al. (2020) | Iran | JA | Mothers | 100 | AR | 3-6 |
| Santiago (2019) | USA | DD | Mothers | 91 | ASD | 5-10 |
| Semke et al. (2010) | USA | JA | Mothers | 89 | BP | 5-9 |
| Seymour et al. (2015) | Australia | JA | Mothers | 100 | LR | 0-1 |
| Shackell (2011) | Canada | MT | NR | --- | DD | 3-12 |
| Shamash (2011) | USA | DD | Mothers | 89 | ASD | 2-6 |
| Shine (2014) | Canada | MT | Mothers | 100 | BP | 4-7 |
| Shoshani and Yaari (2022) | Israel | JA | Mothers | 55 | LR | 2-12 |
| Taraban et al. (2017) Sample 1 | USA | JA | Mothers | 91 | AR | 2-3 |
| Taraban et al. (2017) Sample 2 | USA | JA | Mothers | 91 | AR | 2-3 |
| Taylor et al. (2021) | UK | JA | Mothers | 89 | ASD | 2-11 |
| R. D. Taylor et al. (2012) | USA | JA | Mothers | 100 | BP | 10-18 |
| Z. E. Taylor et al. (2012) | USA | JA | Mothers | 100 | AR | 8-12 |
| Tichovolsky et al. (2013) | USA | JA | Mothers | 95 | BP | 3-5 |
| Tobing (2004) | USA | DD | Mothers | 100 | DD | 2-18 |
| Trivette and Dunst (1992) | USA | JA | Mothers | 100 | DD | <1-5 |

**Table 1,** continued.

| **Study** | **Country** | **Type of Report** | **Primary Sample** | **% of Sample** | **Child Condition** | **Child Age (Years)** |
|---|---|---|---|---|---|---|
| Wade et al. (2008) | USA | JA | Mothers | 96 | DD | 3-7 |
| Weiss and Lunsky (2011) | Canada | JA | Mothers | 91 | ASD | 4-24 |
| Whittaker et al. (2011) | USA | JA | Mothers | 100 | AR | <1-3 |
| Winstone et al. (2021) | USA | JA | Mothers | 100 | AR | 1-3 |
| Wood (2012) | USA | DD | Mothers | 50 | AR | 12-20 |
| Woods (2011) | USA | DD | Mothers | 100 | MC | <1-3 |
| Yeung and Chan (2010) | Hong Kong | JA | Mothers | 80 | AR | 5-9 |
| Yurdusen et al. (2013) | Turkey | JA | Mothers | 100 | BP | 1-6 |

NOTES. The percentage of primary study participants and child age range were estimated in some studies based on information included in the research reports. NR = Not reported or insufficient information was provided to estimate child age range.

[a]BC = Book chapter, BK = Book, CP = Conference presentation, DD = Doctoral dissertation, HT = Honors thesis, JT = Peer-reviewed journal article, MA = Master's thesis, and RR = Unpublished research report.

[b]GM = Grandmothers.

[c]AR = At-risk for poor outcomes, ASD = Autism spectrum disorders, BP = Behavior problems (includes emotional disturbances), DD = Developmental disabilities (includes ADHD, brain injury, cerebral palsy, Down syndrome, hearing loss, intellectual disabilities, and motor delays), LR = Low-risk or no risk for poor outcomes, and MC = Medical conditions (includes asthma, cancer, cocaine-exposed, congenital heart disease, diabetes, epilepsy, leukemia, prematurity, and Smith-Magenis Syndrome).

**Table 2**
Examples of the Scales for Measuring Parents' Psychological Health and Parenting Quality

| **Psychological Health Measures** | |
|---|---|
| **General Health** | **Source** |
| Brief Symptom Inventory* | Derogatis (1993) |
| Depression Anxiety Stress Scale | Lovibond and Lovibond (1995) |
| General Health Questionnaire | Goldberg (1978) |
| Profile of Mood States Scale | Loor and McNair (1988) |
| Symptom Checklist-90-R | Derogatis (1992) |
| **Depression** | |
| Beck Depression Scale | Beck et al. (1961) |
| Brief Symptom Inventory-Depression Subscale | Derogatis (1993) |
| Center for Epidemiological Studies Depression Scale* | Radloff (1977) |
| Depression Anxiety Stress Scale-Stress Subscale | Lovibond and Lovibond (1995) |
| **Personal Stress** | |
| Brief Family Distress Scale | Weiss and Lunsky (2011) |
| Family Stress Scale | Quittner et al. (1991) |
| Perceived Stress Scale* | Cohen et al. (1983) |
| **Well-Being** | |
| Affect Balance Scale | Bradburn (1969) |
| Life Orientation Test | Scheier et al. (1994) |
| Satisfaction with Life Scale* | Diener et al. (1985) |
| World Health Organization Quality of Life Scale | World Health Organization (1996) |
| **Parenting Stress** | |
| Parental Stress Scale | Berry and Jones (1995) |
| Parenting Stress Index* | Abidin (1997) |
| Questionnaire on Resources and Stress (QRS) | Holroyd (1974) |
| Questionnaire on Resources and Stress-Short Form* | Friedrich et al. (1983) |
| Stress Index for Parents | Sheras et al. (1998) |
| **Caregiving Burden** | |
| Caregiver Strain Questionnaire | Brannan et al. (1997) |
| Daily Parenting Hassles Scale | Crnic and Greenberg (1990) |
| Impact on Family Scale* | Stein and Jessop (2003) |
| QRS-Time Demands Subscale | Holroyd (1974) |
| Strengths and Difficulties Questionnaire-Difficulties Subscale | Goodman (1997) |
| **Parenting Quality Measures** | |
| **Parenting Beliefs** | |
| Early Intervention Parenting Self-Efficacy Scale | Guimond et al. (2008) |
| Parent Efficacy for Child Success Scale | Hoover-Dempsey and Sandler (1997) |
| Parental Efficacy Beliefs Scale | Caprara et al. (2006) |
| Parental Locus of Control Scale | Campis et al. (1986) |
| Parental Self-Efficacy Scale | Desjardin (2003) |
| Parenting Sense of Competence Scale* | Gibaud-Wallston and Wandersman (2001) |
| **Parent Involvement** | |
| Alabama Parenting Questionnaire-Involvement Subscales | Shelton et al. (1996) |
| Child Routines Inventory | Sytsma et al. (2001) |
| Family Involvement Questionnaire* | Fantuzzo et al. (2000) |
| Family Routines Inventory | Jensen et al. (1983) |
| Home Observation of Measurement of the Environment (HOME)* | Caldwell and Bradley (1984) |
| Parent-Child Play Scale | Dunst (1986) |
| **Parenting Behavior and Practices** | |
| Alabama Parenting Scale* | Shelton et al. (1996) |
| Caregiver Styles of Interaction Scale | Dunst (2007) |
| Child-Parent Relationship Scale | Driscoll and Pianta (2011) |
| HOME Warmth Subscale | Caldwell and Bradley (1984) |
| Nursing Child Assessment Teaching Scale | Barnard (1978) |

| **Table 2, continued.** | |
|---|---|
| **Parenting Behaviors and Practices,** continued. | Source |
| Parent Behavior Inventory | Lovejoy et al. (1999) |
| Parent-Child Interaction Rating Scales | Sosinsky et al. (2004) |
| Parent-Child Relationship Inventory | Girard (1994) |
| Parenting Styles and Dimensions Questionnaire | Robinson et al. (2001) |

*Indicates the most frequently used scales for the different psychological health and parenting quality constructs.

**Table 3**
**Meta-Essentials Excel Spreadsheet for the Relationships Between Parents' Psychological Health and Parenting Beliefs**

| # | Study name | Include study | Correlation | Sample size | Sufficient data | Subgroup |
|---|---|---|---|---|---|---|
| Caregiving Burden | | | | | | |
| 1 | Almand (2004) | Yes | -0.23 | 64 | Yes | belburd |
| 2 | Biehler et al. (2019) | Yes | -0.39 | 246 | Yes | belburd |
| 3 | Biship (2016) | Yes | -0.27 | 69 | Yes | belburd |
| 4 | Chairinkam et al. (2021) | Yes | -0.33 | 90 | Yes | belburd |
| 5 | Ellis (2019) | Yes | -0.38 | 18 | Yes | belburd |
| 6 | Gartstein & Sheeber (2004) | Yes | -0.42 | 69 | Yes | belburd |
| 7 | Karlioglu & Sari (2019) | Yes | -0.38 | 100 | Yes | belburd |
| 8 | Mahmound et al. (2022) | Yes | -0.37 | 200 | Yes | belburd |
| 9 | Rodenburg et al. (2007) | Yes | -0.43 | 91 | Yes | belburd |
| 10 | Rudelli et al. (2021) | Yes | -0.22 | 63 | Yes | belburd |
| 11 | Shamish (2011) | Yes | -0.41 | 72 | Yes | belburd |
| 12 | Winstone et al. (2011) | Yes | -0.25 | 322 | Yes | belburd |
| Depression | | | | | | |
| 13 | Foster ert al. (2010) Mothers | Yes | -0.46 | 97 | yes | beldep |
| 14 | Foster et al. (2010) Fathers | Yes | -0.34 | 15 | yes | beldep |
| 15 | Garcia-Lopez (2016) Mothers | Yes | -0.40 | 76 | yes | beldep |
| 16 | Garcia-Lopez (2016) Fathers | Yes | -0.46 | 76 | yes | beldep |
| 17 | Kim (2007) | Yes | -0.49 | 408 | yes | beldep |
| 18 | Lee et al. (2009) | Yes | -0.25 | 290 | yes | beldep |
| 19 | Mahmoud et al. (2022) | Yes | -0.32 | 200 | yes | beldep |
| 20 | Palermo et al. (2017) | Yes | -0.47 | 714 | yes | beldep |
| 21 | Rodenburg et al. (2007) | Yes | -0.31 | 91 | yes | beldep |
| 22 | Yurdusen et al. (2013) | Yes | -0.46 | 204 | yes | beldep |
| General Health | | | | | | |
| 23 | Bishop (2016) | Yes | -0.34 | 69 | yes | belgh |
| 24 | Cantwell et al. (2014) | Yes | -0.26 | 167 | yes | belgh |
| 25 | Dissanayake et al. (2020) | Yes | -0.33 | 58 | yes | belgh |
| 26 | Gaoet al. (2012) | Yes | -0.29 | 194 | yes | belgh |
| 27 | Lindsey & Barry (2018) | Yes | -0.20 | 157 | yes | belgh |
| 28 | Mitchell (2015) | Yes | -0.36 | 52 | yes | belgh |
| 29 | Piehleret al. (2014) | Yes | -0.30 | 246 | yes | belgh |
| Parenting Stress | | | | | | |
| 30 | Cekic & Karageyik (2021) | Yes | -0.37 | 492 | yes | belpsi |
| 31 | Chen (2022) | Yes | -0.37 | 263 | Yes | belpsi |
| 32 | Chiel (2018) | Yes | -0.38 | 42 | Yes | belpsi |
| 33 | Collins-Allen (2006) | Yes | -0.41 | 48 | Yes | belpsi |
| 34 | Ehrlick (2004) | Yes | -0.37 | 40 | Yes | belpsi |
| 35 | Garcia-Lopez (2016) Mothers | Yes | -0.32 | 76 | Yes | belpsi |
| 36 | Garcia-Lopez (2016) Fathers | Yes | -0.36 | 76 | Yes | belpsi |
| 37 | Kim (2007) | Yes | -0.50 | 408 | Yes | belpsi |
| 38 | Palermo et al. (2017) | Yes | -0.52 | 714 | Yes | belpsi |
| 39 | Piehler et al. (2014) | Yes | -0.34 | 246 | Yes | belpsi |
| 40 | Raisanen (2013) | Yes | -0.38 | 141 | Yes | belpsi |
| 41 | Semke et al. (2010) | Yes | -0.59 | 207 | Yes | belpsi |

| Table 3, continued. | | | | | | |
|---|---|---|---|---|---|---|
| # | Study name | Include study | Correlation | Sample size | Sufficient data | Subgroup |
| Parenting Stress, continued. | | | | | | |
| 42 | Shine (2014) | Yes | -0.30 | 28 | Yes | belpsi |
| 43 | Tobing (2004) | Yes | -0.52 | 97 | Yes | belpsi |
| 44 | Woods (2011) | Yes | -0.51 | 72 | Yes | belpsi |
| 45 | Yeung & Chan (2010) | Yes | -0.45 | 504 | Yes | belpsi |
| Personal Stress | | | | | | |
| 46 | Cantwell et al. (2014) | Yes | -0.47 | 167 | yes | belstres |
| 47 | Cejas etal. (2021) | Yes | -0.45 | 188 | yes | belstres |
| 48 | Foster et al. (2010) Mothers | Yes | -0.25 | 97 | yes | belstres |
| 49 | Foster et al. (2010) Fathers | Yes | -0.42 | 15 | yes | belstres |
| 50 | Garcia-Lopez (2016) Mothers | Yes | -0.35 | 76 | yes | belstres |
| 51 | Garcia-Lopez (2016) Fathers | Yes | -0.28 | 76 | yes | belstres |
| 52 | Jandric & Kurtovic (2021) | Yes | -0.29 | 107 | yes | belstres |
| 53 | Robinson (2019) | Yes | -0.44 | 249 | yes | belstres |
| 54 | Shamash (2011) | Yes | -0.44 | 72 | yes | belstres |
| 55 | Tobing (2004) | Yes | -0.35 | 97 | yes | belstres |
| 56 | Yurdusen et al. (2013) | Yes | -0.41 | 204 | yes | belstres |
| Well-Being | | | | | | |
| 57 | Dubriwny et al. (2010) | Yes | 0.31 | 156 | yes | belwb |
| 58 | Foster et al. (2010) Mothers | Yes | 0.43 | 97 | yes | belwb |
| 59 | Garcia-Lopez (2016) Mothers | Yes | 0.50 | 76 | yes | belwb |
| 61 | Garcia-Lopez (2016) Fathers | Yes | 0.41 | 76 | yes | belwb |
| 62 | Quinn (2017) | Yes | 0.34 | 96 | yes | belwb |
| 63 | Shackell (2011) | Yes | 0.32 | 30 | yes | belwb |
| 64 | Shoshani (2022) | Yes | 0.38 | 832 | yes | belwb |
| 65 | Taylor et al. (2021) | Yes | 0.58 | 200 | yes | belwb |

**Table 4**
**Meta-Essentials Excel Spreadsheet for the Relationships Between Parents' Psychological Health and Parental Involvement**

| # | Study name | Include study | Correlation | Sample size | Sufficient data | Subgroup | Involvement Setting |
|---|---|---|---|---|---|---|---|
| Caregiving Burden | | | | | | | |
| 1 | Benson (2015) | Yes | -0.27 | 113 | yes | invburd | School |
| 2 | Benson (2015) | Yes | -0.26 | 113 | yes | invburd | Home |
| 3 | Comfort (1987) Mothers | Yes | -0.21 | 24 | yes | invburd | Home |
| 4 | Comfort (1987) Fathers | Yes | -0.19 | 24 | yes | invburd | Home |
| 5 | Dinehart et al. (2006) | Yes | -0.28 | 56 | yes | invburd | Home |
| 6 | Santiago (2019) | Yes | -0.12 | 68 | yes | invburd | School |
| Depression | | | | | | | |
| 7 | Brody & Flor (1997) | Yes | -0.16 | 156 | yes | invdep | Home |
| 8 | Equihua (2010) | Yes | -0.18 | 43 | yes | invdep | School |
| 9 | Gavidia-Payne (1997) M | Yes | -0.26 | 75 | yes | invdep | School |
| 10 | Gavidia-Payne (1997) F | Yes | -0.17 | 67 | yes | invdep | School |
| 11 | Huang et al. (2018) | Yes | -0.18 | 262 | yes | invdep | School |
| 12 | Kohl et al. (2000) | Yes | -0.29 | 331 | yes | invdep | School |
| 13 | Kohl et al. (2000) | Yes | -0.28 | 331 | yes | invdep | Home |
| 14 | Lee et al. (2009) | Yes | -0.12 | 290 | yes | invdep | Home |
| 15 | Nievar et al. (2008) | Yes | -0.21 | 96 | yes | invdep | Home |
| 16 | Okado et al. (2014) | Yes | -0.11 | 117 | yes | invdep | Home |
| 17 | Okado et al. (2014) | Yes | -0.18 | 117 | yes | invdep | School |
| 18 | Robokos (2007) | Yes | -0.16 | 1807 | yes | invdep | Home |
| 19 | Sajedi et al. (2020) | Yes | -0.18 | 1067 | yes | invdep | Home |
| General Health | | | | | | | |
| 20 | Arnold et al. (2008) | Yes | -0.15 | 163 | yes | invgh | School |
| 21 | Garland et al. (2013) | Yes | -0.20 | 153 | yes | invgh | Home |
| 22 | Huang et al. (2018) | Yes | -0.14 | 262 | yes | invgh | School |
| 23 | Jordan (2003) | Yes | -0.28 | 153 | yes | invgh | Home |
| 24 | Kelley et al. (2011) | Yes | -0.17 | 230 | yes | invgh | Home |
| 25 | McRae et al. (2020) | Yes | -0.13 | 300 | yes | invgh | Home |
| 26 | Seymour et al. (2015) | Yes | -0.25 | 224 | yes | invgh | Home |
| 27 | Tichovolsky et al. (2013) | Yes | -0.13 | 129 | yes | invgh | School |
| Parenting Stress | | | | | | | |
| 28 | Cardenas (2020) | Yes | -0.14 | 632 | yes | invpsi | Home |
| 29 | Collins-Allen (2006) | Yes | -0.15 | 48 | yes | invpsi | School |
| 31 | Glenn et al. (2008) | Yes | -0.25 | 80 | yes | invpsi | Home |
| 32 | Okado et al. (2014) | Yes | -0.17 | 117 | yes | invpsi | Home |
| 33 | Okado et al. (2014) | Yes | -0.20 | 117 | yes | invpsi | School |
| 34 | Robokos (2007) | Yes | -0.24 | 1807 | yes | invpsi | Home |
| 35 | Semke et al. (2010) | Yes | -0.20 | 207 | yes | invpsi | Home |
| 36 | Semke et al. (2010) | Yes | -0.20 | 207 | yes | invpsi | School |
| 37 | Unger et al. (2001) | Yes | -0.20 | 104 | yes | invpsi | School |
| 38 | Woods (2011) | Yes | -0.26 | 72 | yes | invpsi | Home |
| Personal Stress | | | | | | | |
| 39 | Cejas et al. (2021) | Yes | -0.32 | 188 | yes | invstres | Home |
| 40 | Equihua (2001) | Yes | -0.17 | 43 | yes | invstres | School |
| 41 | Jordan (2003) | Yes | -0.25 | 153 | yes | invstres | Home |

| Table 4, continued. | | | | | | | |
|---|---|---|---|---|---|---|---|
| Personal Stress, continued. | | | | | | | |
| 42 | Kelley et al. (2011) | Yes | -0.17 | 230 | yes | invstres | Home |
| 43 | Lee et al. (2009) | Yes | -0.32 | 290 | yes | invstres | Home |
| 44 | McRae et al. (2020) | Yes | -0.13 | 300 | yes | invstres | Home |
| 45 | Sajedi et al. (2020) | Yes | -0.11 | 1067 | yes | invstres | Home |
| Well-Being | | | | | | | |
| 46 | Cardenas (2020) | Yes | 0.24 | 632 | yes | invwb | School |
| 48 | Huang et al. (2018) | Yes | 0.14 | 262 | yes | invwb | School |
| 49 | Richter et al. (2018) | Yes | 0.25 | 291 | yes | invwb | Home |
| 50 | Shoshani & Yaari (2022) | Yes | 0.38 | 832 | yes | invwb | Home |
| 51 | Z. E. Taylor et al. (2012) | Yes | 0.30 | 674 | yes | invwb | Home |

**Table 5**
**Meta-Essentials Excel Spreadsheet for the Relationships Between Parents' Psychological Health and Parenting Behavior and Practices**

| # | Study name | Include study | Correlation | Sample size | Sufficient data | Subgroup |
|---|---|---|---|---|---|---|
| Caregiving Burden | | | | | | |
| 1 | Comfort (1987) Fathers | Yes | -0.39 | 24 | Yes | pbpburd |
| 2 | Dinehart et al. (2006) | Yes | -0.28 | 56 | Yes | pbpburd |
| 3 | Fenning et al. (2014) Mothers | Yes | -0.18 | 61 | Yes | pbpburd |
| 4 | Fenning et al. (2014) Fathers | Yes | -0.21 | 49 | Yes | pbpburd |
| 5 | Fiese et al. (2008) | Yes | -0.27 | 60 | Yes | pbpburd |
| 6 | Frank et al. (2017) | Yes | -0.47 | 979 | Yes | pbpburd |
| 7 | Kilmer et al. (2010) | Yes | -0.22 | 100 | Yes | pbpburd |
| 8 | Platt et al. (2014) Fathers | Yes | -0.33 | 150 | Yes | pbpburd |
| 9 | Platt et al. (2014) Mothers | Yes | -0.21 | 188 | Yes | pbpburd |
| 10 | Rodenburg et al. (2007) | Yes | -0.46 | 91 | Yes | pbpburd |
| 11 | Weiss & Lunsky (2011) | Yes | 0.26 | 164 | Yes | pbpburd |
| Depression | | | | | | |
| 12 | Baker & Iruka (2013) | Yes | -0.21 | 246 | Yes | pbpdep |
| 13 | Biondic (2019) Mothers | Yes | -0.38 | 61 | Yes | pbpdep |
| 14 | Biondic (2019) Fathers | Yes | -0.06 | 61 | Yes | pbpdep |
| 15 | Brody et al. (2006) | Yes | -0.23 | 172 | Yes | pbpdep |
| 16 | Dahl (1993) | Yes | -0.19 | 61 | Yes | pbpdep |
| 17 | Dunst (1985) Study 3 | Yes | -0.20 | 59 | Yes | pbpdep |
| 18 | Dunst (1985) Study 4 | Yes | -0.28 | 64 | Yes | pbpdep |
| 19 | Dunst (1988) | Yes | -0.26 | 73 | Yes | pbpdep |
| 20 | Emmen et al. (2013) | Yes | -0.29 | 107 | Yes | pbpdep |
| 21 | Huang et al. (2017) | Yes | -0.34 | 303 | Yes | pbpdep |
| 22 | Huang et al. (2018) | Yes | -0.17 | 262 | Yes | pbpdep |
| 23 | Lee et al. (2009) | Yes | -0.23 | 290 | Yes | pbpdep |
| 24 | Letiecq & Kobinsky (2003) | Yes | -0.32 | 61 | Yes | pbpdep |
| 25 | Okado et al. (2014) | Yes | -0.38 | 117 | Yes | pbpdep |
| 26 | Robokos (2007) | Yes | -0.12 | 1807 | yes | pbpdep |
| 27 | Rodenburg et al. (2007) | Yes | -0.31 | 91 | Yes | pbpdep |
| 28 | Taradan et al. (2017) Sample 1 | Yes | -0.19 | 526 | Yes | pbpdep |
| 29 | Tichovolskyet al. (2013) | Yes | -0.13 | 129 | Yes | pbpdep |
| 30 | Wade et al. (2008) | Yes | -0.15 | 198 | Yes | pbpdep |
| 31 | Whittaker et al. (2011) | Yes | -0.19 | 114 | Yes | pbpdep |
| 32 | Yurdusen et al. (2013) | Yes | -0.25 | 204 | Yes | pbpdep |
| General Health | | | | | | |
| 33 | Carreras et al. (2019) | Yes | -0.14 | 62 | Yes | pbpgh |
| 34 | Chisholm et al. (2014) | Yes | -0.32 | 49 | Yes | pbpgh |
| 35 | Dissanayake et al. (2020) | Yes | -0.43 | 58 | Yes | pbpgh |
| 36 | Garland et al. (2013) | Yes | -0.12 | 153 | Yes | pbpgh |
| 37 | Jordan (2003) | Yes | -0.27 | 153 | Yes | pbpgh |
| 38 | Merson (2012) | Yes | -0.31 | 177 | Yes | pbpgh |
| 39 | Mitchell (2011) | Yes | -0.40 | 52 | Yes | pbpgh |
| 40 | Piehler et al. (2014) | Yes | -0.36 | 246 | Yes | pbpgh |
| 41 | Seymour et al. (2014) | Yes | -0.50 | 224 | Yes | pbpgh |
| | | | | | | |
| | | | | | | |

| Table 5, continued. | | | | | | |
|---|---|---|---|---|---|---|
| # | Study name | Include study | Correlation | Sample size | Sufficient data | Subgroup |
| Parenting Stress | | | | | | |
| 42 | Baker & Iruka (2013) | Yes | -0.31 | 246 | Yes | pbppsi |
| 43 | Biondic (2019) Mothers | Yes | -0.43 | 61 | Yes | pbppsi |
| 44 | Biondic (2019) Fathers | Yes | -0.35 | 61 | Yes | pbppsi |
| 45 | Bradley et al. (2023) | Yes | -0.37 | 483 | Yes | pbppsi |
| 46 | Cekic & Karageyik (2021) | Yes | -0.35 | 492 | Yes | pbppsi |
| 47 | Chiel (2018) | Yes | -0.38 | 42 | Yes | pbppsi |
| 48 | Cooke (2010) | Yes | -0.40 | 110 | Yes | pbppsi |
| 49 | Mitchell (2011) | Yes | -0.30 | 52 | Yes | pbppsi |
| 50 | Okado et al. (2014) | Yes | -0.29 | 117 | Yes | pbppsi |
| 51 | Robokos (2007) | Yes | -0.18 | 1807 | Yes | pbppsi |
| 52 | Rogers et al. (2009) | Yes | -0.20 | 103 | Yes | pbppsi |
| 53 | Unger et al. (2011) | Yes | -0.11 | 104 | Yes | pbppsi |
| 54 | Whittaker et al. (2011) | Yes | -0.40 | 114 | Yes | pbppsi |
| Personal Stress | | | | | | |
| 55 | Anderson (2015) | Yes | -0.52 | 127 | Yes | pbpstres |
| 56 | Kilme et al.r (2010) | Yes | -0.35 | 100 | Yes | pbpstres |
| 57 | R. D. Taylor et al. (2012) | Yes | -0.32 | 200 | Yes | pbpstres |
| 58 | Weiss &Lunsky (2011) | Yes | 0.26 | 164 | Yes | pbpstres |
| 59 | Wood (2012) | Yes | -0.25 | 146 | Yes | pbpstres |
| 60 | Yurdusen et al. (2013) | Yes | -0.31 | 204 | Yes | pbpstres |
| Well-Being | | | | | | |
| 61 | Bax (2005) Mothers | Yes | 0.27 | 130 | Yes | pbpwb |
| 62 | Bax (2005) Fathers | Yes | 0.45 | 130 | Yes | pbpwb |
| 63 | Cooke (2010) | Yes | 0.17 | 110 | Yes | pbpwb |
| 64 | Dunst (1985) Study 5 | Yes | 0.15 | 63 | Yes | pbpwb |
| 65 | Dunst (1985) Study 6 | Yes | 0.17 | 103 | Yes | pbpwb |
| 66 | Huang et al. (2018) | Yes | 0.13 | 262 | Yes | pbpwb |
| 67 | Podjarny (2007) | Yes | 0.31 | 18 | Yes | pbpwb |
| 67 | Z. E. Taylor et al. (2012) | Yes | 0.11 | 674 | Yes | pbpwb |
| 69 | Trivette & Dunst (1992) | Yes | 0.30 | 82 | Yes | pbpwb |

References

Abidin, R. R. (1997). Parenting Stress Index: A measure of the parent-child system. In C. P. Zalaquett & J. Wood (Eds.), *Evaluating stress: A book of resources* (pp. 277-291). Scarecrow Education.

Almand, C. S. (2004). *Parenting daily hassles and parents of children with disabilities: Relationships to maternal efficacy, maternal satisfaction, and social support*. [Master's Thesis, University of Georgia]. http://www.fcs.uga.edu/ss/theses.html

Anderson, R. E. (2015). Focusing on family: Parent-child relationships and school readiness among economically impoverished black children. *Journal of Negro Education*, *84*(3), 442-456. https://doi.org/10.7709/jnegroeducation.84.3.0442

Arnold, D. H., Zeljo, A., Doctoroff, G. L., & Ortiz, C. (2008). Parental involvement in preschool: Predictors and the relation of involvement to preliteracy development. *School Psychology Review*, *37*(1), 74-90. https://doi.org/10.1080/02796015.2008.12087910

Baker, C. E., & Iruka, I. U. (2013). Maternal psychological functioning and children's school readiness: The mediating role of home environment for African American children. *Early Childhood Research Quarterly*, *28*, 509-519. https://doi.org/10.1016/j.ecresq.2013.02.004

Barnard, K. E. (1978). *Nursing Child Assessment Teaching Scale*. School of Nursing, University of Washington, Seattle, WA.

Bax, K. A. (2005). *Between parent similarities in child-rearing goals: Relations to parental, marital and individual adult well-being*. [Doctoral Dissertation, University of Ottawa]. https://ruor.uottawa.ca/bitstream/10393/29196/1/NR10947.PDF

Beck, A. T., Ward, C. H., Mendelson, M., Mock, J., & Erbaugh, J. (1961). An inventory for measuring depression. *Archives of General Psychiatry*, *4*, 561-571. https://doi.org/510.1001/archpsyc.1961.01710120031004.

Benson, R. R. (2015). Longitudinal effects of educational involvement on parent and family functioning among mothers of children with ASD. *Research in Autism Spectrum Disorders*, *11*, 42-55. https://doi.org/10.1016/j.rasd.2014.11.011

Berry, J. O., & Jones, W. H. (1995). The Parental Stress Scale: Initial evidence. *Journal of Social and Personal Relationships*(12), 463-472. https://doi.org/10.1177/0265407595123009

Biondic, D. (2019). *Parenting stress and parenting behaviors of parents of adolescents with attention-deficit/hyperactivity disorder*. [Doctoral Dissertation, University of Toronto]. https://tspace.library.utoronto.ca/bitstream/1807/97332/1/Biondic_Daniella_%20_201911_PhD_thesis.pdf

Bishop, M. (2016). *Stress appraisal, coping resources, and psychological functioning in parents of infants and toddlers diagnosed with congenital heart disease*. [Master's Thesis, Georgia State University]. https://doi.org/10.57709/10044187

Bradburn, N. M. (1969). *The structure of psychological well-being*. Aldine.

Bradley, R. S., Staples, G. L., Quetsch, L. B., Aloia, L. S., Brown, C. E., & Kanne, S. M. (2023). Associations between parenting stress and quality of time in families of youth with Autism Spectrum Disorder. *Journal of Autism and Developmental Disorders*, *10*, 1-12. https://doi.org/10.1007/s10803-022-05852-0

Brannan, A. M., Heflinger, C. A., & Bickman, L. (1997). The Caregiver Strain Questionnaire: Measuring the impact on the family living with a child with serious emotional disturbances. *Journal of Emotional and Behavioral Disorders*, *5*(4), 212-222. https://doi.org/10.1177/106342669700500404

Broderick, C. B. (1993). *Understanding family process: Basics of family systems theory*. Sage Publications.

Brody, G. H., & Flor, D. L. (1997). Maternal psychological functioning, family processes, and child adjustment in rural, single-parent, African American families. *Developmental Psychology*, *33*(6), 1000-1011. https://doi.org/10.1037//0012-1649.33.6.1000

Brody, G. H., Murry, V. M., Chen, Y.-F., Kogan, S. M., & Brown, A. C. (2006). Effects of family risk factors on dosage and efficacy of a family-centered preventive intervention for rural African Americans. *Prevention Science*, *7*, 281-291. https://doi.org/10.1007/s11121-066-0032-7

Bronfenbrenner, U. (1979). *The ecology of human development: Experiments by nature and design*. Harvard University Press.

Caldwell, B. M., & Bradley, R. H. (1984). *Home Observation for Measurement of the Environment*. Unpublished scale, University of Arkansas at Little Rock.

Campis, L. K., Lyman, R. D., & Prentice-Dunn, S. (1986). The parental locus of control scale: Development and validation. *Journal of clinical child psychology*, *15*, 260-267. https://doi.org/210.1207/s15374424jccp15371503_15374410.

Cantwell, J., Muldoon, O., & Gallagher, S. (2014). Social support and mastery influence the association between stress and poor physical health in parents caring for children with developmental disabilities. *Research in Developmental Disabilities*, *35*(9), 2215-2223. https://doi.org/10.1016/j.ridd.2014.05.012

Caprara, G. V., Regalia, C., Scabini, E., Barbanelli, C., & Bandura, A. (2006). Assessment of filial, parental, marital, and collective family efficacy beliefs. *European Journal of Psychological Assessment*, *20*(4), 246-266. https://doi.org/10.1027/1015-5759.20.4.247

Cardenas, J. E. F. (2020). *Social support from childcare providers and maternal well-being: Finding relationships that matter*. [Doctoral Dissertation, Texas Tech University]. https://ttu-ir.tdl.org/bitstream/handle/2346/85855/FYALL-DISSERTATION-2020.pdf?sequence=1

Carreras, J., Carter, A. S., Heberle, A., Forbes, D., & Gray, S. A. O. (2019). Emotional regulation and parent distress: Getting at the heart of sensitive parenting among parents of preschool children experiencing high sociodemographic risk. *Journal of Child and Family Studies*, *28*, 2953-2962. https://doi.org/0.1007/s10826-019-01471-z

Cejas, I., Mitchell, C. M., Barker, D. H., Sarangoulis, C., Eisenberg, L. S., & Quittner, A. L. (2021). Parenting stress, self-efficacy, and involvement: Effects on spoken language ability three years after cochlear implantation. *Otology & Neurotology*, *42*(S10), S11-S18. https://doi.org/10.1097/MAO.0000000000003374

Cekic, A., & Karageyik, K. (2021). Analyzing parenting stress in terms of parental self-efficacy and parent-child communication. *International Journal of Education, Psychology and Counselling*, *6*(40), 14-34. https://doi.org/10.35631/IJEPC.640002

Chairinkam, S., Pothiban, L., Mesukko, J., & Niyomkar, S. (2021). Factors predicting maternal caregiving behaviours for leukemic children undergoing chemotherapy. *Suranaree Journal of Science and Technology*, *29*(4), 1-13. http://cmuir.cmu.ac.th/jspui/handle/6653943832/74924

Chen, Y., Cheng, T. L., & Lv, F. (2022). Sense of parenting efficacy, perceived family interactions, and parenting stress among mothers of children with Autism Spectrum Disorders. *Frontiers in Psychology*, *13*, Article 878158. https://doi.org/10.3389/fpsyg.2022.878158

Chiel, Z. A. (2018). *Parental attributions of control and self-efficacy: Observed parenting behaviors in mothers of preschool children with Autism Spectrum Disorder*. [Doctoral Dissertation, Columbia University]. https://doi.org/10.7916/D8CR7B2Q

Chisholm, V., Gonzalez, A., & Atkinson, L. (2014). Interpersonal engagement mediates the relation between maternal affect and externalizing behaviour in young children with Type 1 Diabetes. *PLoS ONE*, *9*(6), Article e97672. https://doi.org/10.1371/journal.pone.0097672

Cohen, S. H., Kamarck, T., & Mermelstein, R. (1983). A global measure of perceived stress. *Journal of Health and Social Behavior*, *24*(4), 385-396. https://doi.org/10.2307/2136404

Collins-Allen, S. (2006). *Maternal involvement and professional support as predictors of maternal self-efficacy*. [Doctoral Dissertation, St. John's University]. https://www.proquest.com/psychology/docview/305271626/fulltextPDF/C0EDA1B8D02F4DD2PQ/2?accountid=8385

Comfort, M. (1987). *Differential relationships of parental perceptions to maternal and paternal involvement in play with young high risk and handicapped children*. Educational Resource Information Center. https://files.eric.ed.gov/fulltext/ED288322.pdf

Cooke, J. E. (2010). *Hope, optimism, stress, and social support in parents of children with intellectual disabilities*. [Doctoral Dissertation, University of Southern Mississippi]. https://aquila.usm.edu/cgi/viewcontent.cgi?article=2006&context=dissertations

Crnic, K. A., & Greenberg, M. T. (1990). Minor parenting stresses with young children. *Child Development*, *61*, 1628-1637. https://doi.org/1610.2307/1130770.

Dahl, R. W. (1993). *Maternal discipline approaches: A comparison between children with conduct problems and a nonclinic group*. [Doctoral Dissertation, University of Washington]. https://www.proquest.com/docview/304094933?pq-origsite=gscholar&fromopenview=true

Derogatis, L. R. (1992). *SCL-90-R: Administration, scoring, and procedures manual-II for the Revised Rating Scale* (2nd ed.). Clinical Psychometric Research.

Derogatis, L. R. (1993). *Brief Symptom Inventory*. Pearson Assessment.

Desjardin, J. L. (2003). Assessing parental perceptions of self-efficacy and involvement in families of young children with hearing loss. *Volta Review*, *103*, 391-409.

Diener, E., Emmons, R. A., Larsen, R. J., & Griffin, S. (1985). The Satisfaction With Life Scale. *Journal of Personality Assessment*, *49*, 71-75. https://doi.org/10.1207/s15327752jpa4901_13

Dinehart, L. H. B., Dice, J. L., Dobbins, D. R., Claussen, A. H., & Bono, K. E. (2006). Proximal variables in families of children prenatally exposed to cocaine and enrolled in a center- or home-based intervention. *Journal of Early Intervention*, *29*, 32-46. https://doi.org/10.1177/105381510602900103

Dissanayake, C., Richdale, A., Kolivaas, N., & Pamment, L. (2020). An exploratory study of Autism traits and parenting. *Journal of Autism and Developmental Disorders*, *50*(7), 2593-2606. https://doi.org/10.1007/s10803-019-03984-4

Driscoll, K., & Pianta, R. C. (2011). Mothers' and fathers' perceptions of conflict and closeness in parent-child relationships during early childhood. *Journal of Early Childhood and Infant Psychology*, *7*, 1-24.

Dubriwny, N., & Hellman, C. M. (2010). *The impact of program services on parent-child interaction and hope*. University of Oklahoma Schusterman Center. https://www.parentchildcenter.org/wp-content/uploads/2012/03/PCCT-Report.pdf

Dunst, C. J. (1985). Rethinking early intervention. *Analysis and Intervention in Developmental Disabilities*, *5*, 165-201. https://doi.org/10.1016/S0270-4684(85)80012-4

Dunst, C. J. (1986). *Parent-Child Play Scale: A rating scale for assessing parent-child play opportunities*. Winterberry Press.

Dunst, C. J. (2000). Revisiting "Rethinking early intervention". *Topics in Early Childhood Special Education*, *20*, 95-104. https://doi.org/110.1177/027112140002000205.

Dunst, C. J. (2007). *Caregiver styles of interaction scales: Revised and updated*. Winterberry Press.

Dunst, C. J. (2017). Family systems early childhood intervention. In H. Sukkar, C. J. Dunst, & J. Kirkby (Eds.), *Early childhood intervention: Working with families of young children with special needs* (pp. 38-60). Routledge.

Dunst, C. J. (2020). Modeling the relationships between parent strengths, parenting efficacy beliefs, and child social-affective behavior. *International Journal of Child Development and Mental Health*, *8*(2), 11-18. https://he01.tci-thaijo.org/index.php/cdmh/article/view/232866

Dunst, C. J. (2021a). Family hardiness and parent and family functioning in households with children experiencing adverse life events: A meta-analysis. *International Journal of Psychological Research*, *14*(2), 93-118. https://doi.org/10.21500/20112084.5236

Dunst, C. J. (2021b). Meta-analysis of the relationships between the adequacy of family resources and parenting beliefs and practices. *British Journal of Psychology Research*, *9*(2), 56-76. https://www.eajournals.org/journals/british-journal-of-psychology-research-bjpr/vol-9-issue-2-2021/meta-analysis-of-the-relationships-between-the-adequacy-of-family-resources-and-parenting-beliefs-and-practices/

Dunst, C. J. (2021c). Meta-analysis of the relationships between the adequacy of family resources and personal, family, and child well-being. *European Journal of Psychological Research*, *8*(3), 35-49. www.eajournals.org/journals/british-journal-of-psychology-research-bjpr/vol-9-issue-2-2021/meta-analysis-of-the-relationships-between-the-adequacy-of-family-resources-and-parenting-beliefs-and-practices/

Dunst, C. J. (2021d). A meta-analytic investigation of the relationships between different dimensions of family strengths and personal and family well-being. *Journal of Family Research*, *33*(1), 209-229. https://doi.org/10.20377/jfr-578

Dunst, C. J. (2022a). Associations between perceived family social support and the psychological health of caregivers of children and adolescents: A systematic review and meta-analysis. *European Journal of Psychological Research*, *9*(2), 32-57. www.idpublications.org/ejpr-vol-9-no-2-2022/

Dunst, C. J. (2022b). Child studies through the lens of applied family social systems theory. *Child Studies*, *1*, 37-64. https://doi.org/10.21814/childstudies.4126

Dunst, C. J. (2022c). Functional social support and psychological health and functioning: A meta-analysis of studies of parents of children and adolescents with identified disabilities. *European Journal of Psychological Health*, *9*(3), 62-82. http://www.idpublications.org/wp-content/uploads/2023/01/Full-Paper-FUNCTIONAL-SOCIAL-SUPPORT-AND-PSYCHOLOGICAL-HEALTH-AND-FUNCTIONING-A-META-ANALYSIS-OF-STUDIES.pdf

Dunst, C. J. (2022d). Systematic review and meta-analysis of family needs studies: Relationships with parent, family and child functioning. *European Journal of Psychology and Educational Research*, *5*(1), 11-32. https://doi.org/10.12973/ejper.5.1.11

Dunst, C. J. (2022e). Systematic review and meta-analysis of the relationships between family social support and parenting stress, burden, beliefs and practices. *International Journal of Health and Psychology Research*, *10*(3), 1-27. https://doi.org/10.37745/ijhpr.13/vol10no1pp.18-30

Dunst, C. J. (2022f). Systematic review and meta-analysis of the relationships between the adequacy of family resources and parenting stress. *International Journal of Health and Psychology Research*, *10*(1), 18-30. https://doi.org/0.37745/ijhpr.13/vol10no1pp.18-30

Dunst, C. J. (2023). Meta-analyses of the relationships between family systems practices, parents' psychological health, and parenting quality. *International Journal of Environmental Research and Public Health*, *20*.

Dunst, C. J., Hamby, D. W., & Brookfield, J. (2007). Modeling the effects of early childhood intervention variables on parent and family well-being. *Journal of Applied Quantitative Methods*, *2*, 268-288. http://www.jaqm.ro/issues/volume-262,issue-263/261_ModelingtheEffects.php

Dunst, C. J., Hamby, D. W., & Raab, M. (2019). Modeling the relationships between practitioner capacity-building practices and the behavior and development of young children with disabilities and delays. *Educational Research and Reviews*, *14*(9), 309-319. https://doi.org/10.5897/ERR2019.3742

Dunst, C. J., Serrano, A. M., Mas, J. M., & Espe-Sherwindt, M. (2021). Meta-analysis of the relationships between family strengths and parent, family and child well-being. *European Journal of Applied Positive Psychology*, *5*, Article 5. https://www.nationalwellbeingservice.org/volumes/volume-5-2021/volume-5-article-5/

Dunst, C. J., & Trivette, C. M. (1988). Determinants of parent and child interactive behavior. In K. Marfo (Ed.), *Parent-child interaction and developmental disabilities: Theory, research, and intervention* (pp. 3-31). Praeger.

Dunst, C. J., & Trivette, C. M. (2009). Meta-analytic structural equation modeling of the influences of family-centered care on parent and child psychological health. *International Journal of Pediatrics*, *2009*, 1-9. http://downloads.hindawi.com/journals/ijped/2009/576840.pdf

Dunst, C. J., Trivette, C. M., & Deal, A. G. (1988). *Enabling and empowering families: Principles and guidelines for practice*. Brookline Books.

Dunst, C. J., Trivette, C. M., & Deal, A. G. (Eds.). (1994). *Supporting and strengthening families: Methods, strategies and practices*. Brookline Books.

Ehrlick, A. L. W. (2004). *Maternal and paternal attributions and perceptions related to parent-child interactions*. [Doctoral Dissertation, Utah State University]. https://digitalcommons.usu.edu/etd/6186/?utm_source=digitalcommons.usu.edu%2Fetd%2F6186&utm_medium=PDF&utm_campaign=PDFCoverPages

Ellis, S. (2019). *Parenting a child with a learning disability: Parent self-efficacy, parent attribution and parent perception of behaviour*. [Doctoral Dissertation, University of Leicester]. https://figshare.le.ac.uk/articles/thesis/Parenting_a_Child_with_a_Learning_Disability_Parent_Self-efficacy_Parent_Attribution_and_Parent_Perception_of_Behaviour/11799849/1

Emmen, R. A. G., Malda, M., Mesman, J., van IJzendoorn, M. H., Prevoo, M. J. L., & Yenuad, N. (2013). Socioeconomic status and parenting in ethnic minority families: Testing a minority family stress model. *Journal of Family Psychology*, *27*(6), 896-904. https://doi.org/10.1037/a0034693

Equihua, D. (2010). *The relationship of mental health and family factors to parental involvement in their children's education among Latino mothers*. [Master's Thesis, California State University, Long Beach]. https://www.proquest.com/docview/756626364?pq-origsite=gscholar&fromopenview=true

Fantuzzo, J., Tighe, E., & Childs, S. (2000). Family Involvement Questionnaire: A multivariate assessment of family participation in early childhood education. *Journal of Educational Psychology*, *92*(2), 367-376. https://doi.org/10.1037/0022-0663.92.2.367

Fenning, R. M., Baker, J. K., & Baker, B. L. (2014). Parent-child interaction over time in families of young children with borderline intellectual functioning. *Journal of Family Psychology*, *28*(3), 326-335. https://doi.org/10.1037/a0036537

Fiese, B. H., Winter, M., Anbar, R., Howell, K., & Poltrock, S. (2008). Family climate of routine asthma care: Associating perceived burden and mother-child interaction patterns and child well-being. *Family Process*, *47*(1), 63-79. https://doi.org/10.1111/j.1545-5300.2008.00239.x

Foster, R. H., Kozachek, S., Stern, M., & Elsea, S. H. (2010). Caring for the caregivers: An investigation of factors related to well-being among parents caring for a child with Smith-Magenis Syndrome. *Journal of Genetic Counselling*, *19*(2), 187-198. https://doi.org/10.1007/s10897-009-9273-5

Frank, S. J., Roubal, K. C., Breitzer, G. M., & Godin, J. L. (2017). Separating the effects of child problems and parent-child interactions on caregiver strain. *Journal of Child and Family Studies*, *26*, 248-261. https://doi.org/10.1007/s10826-016-0560-0

Friedrich, W. N., Greenberg, M. T., & Crnic, K. (1983). A short form of the Questionnaire on Resources and Stress. *American Journal of Mental Deficiency*, *88*, 41-48.

Gao, L.-L., Chan, S. W.-C., & Sun, K. (2012). Effects of an interpersonal-psychotherapy-oriented childbirth education programme for Chinese first-time childbearing women at 3-month follow up: Randomized controlled trial. *International Journal of Nursing Studies*, *49*, 274-281. https://doi.org/10.1016/j.ijnurstu.2011.09.010

Garcia-Lopez, C., Sarria, E., & Pozo, P. (2016). Parental self-efficacy and positive contributions regarding Autism Spectrum condition: An actor-partner interdependence model. *Journal of Autism and Developmental Disorders*, *46*(2), 2385-2398. https://doi.org/10.1007/s10803-016-2771-z

Garland, B. H., Barry, T. D., & Heffer, R. W. (2013). Parenting techniques as a mediator between female caregivers' internalizing symptoms and externalizing behaviors among preschool-aged children. *Head Start Dialog*, *16*(1), 148-171. https://journals.charlotte.edu/dialog/article/view/48

Gartstein, M. A., & Sheeber, L. (2004). Child behavior problems and maternal symptoms of depression: A mediational model. *Journal of Child and Adolescent Psychiatric Nursing*, *17*, 141-150. https://doi.org/10.1111/j.1744-6171.2004.tb00011.x

Gavidia-Payne, S., & Stoneman, Z. (1997). Family predictors of maternal and paternal involvement in programs for young children with disabilities. *Child Development*, *68*, 701-717. https://doi.org/10.1111/j.1467-8624.1997.tb04231.x

Gibaud-Wallston, J., & Wandersman, L. P. (2001). Parenting Sense of Competence Scale. In J. Touliatos, B. F. Perlmutter, & M. A. Straus (Eds.), *Handbook of family measurement techniques: Vol. 3. Instruments and index* (pp. 315-325). Sage Publications.

Girard, A. B. (1994). *Parent-Child Relationship Inventory*. Western Psychological Services.

Glenn, S., Cunningham, C., & Poole, H. (2008). Maternal parenting stress and its correlates in families with a young child with cerebral palsy. *Child: Care, Health and Development*, *35*, 71–78. https://doi.org/10.1111/j.1365-2214.2008.00891.x

Goldberg, D. P. (1978). *Manual of the General Health Questionnaire*. NFER Publishing.

Goodman, R. (1997). The Strengths and Difficulties Questionnaire: A research note. *Journal of Child Psychology and Psychiatry*, *38*, 581-586. https://doi.org/10.1111/j.1469-7610.1997.tb01545.x

Guimond, A. B., Wilcox, M. J., & Lamorey, S. G. (2008). The Early Intervention Parenting Self-Efficacy Scale (EIPSES): Scale construction and initial psychometric evidence. *Journal of Early Intervention*, *30*, 295-320. https://doi.org/10.1177/105381510832081

Holroyd, J. (1974). The Questionnaire on Resources and Stress: An instrument to measure family response to a handicapped family member. *Journal of Community Psychology*, *2*, 92-94. https://doi.org/10.1002/1520-6629(197401)2:1<92::AID-JCOP2290020133>3.0.CO;2-A

Hoover-Dempsey, K. V., & Sandler, H. M. (1997). Why do parents become involved in their children's education? *Review of Educational Research*, *67*, 3-42. https://doi.org/10.3102/00346543067001003

Huang, K.-Y., Abura, G., Theise, R., & Nakigudde, J. (2017). Parental depression and associations with parenting and children's physical and mental health in a sub-Saharan African setting. *Child Psychiatry and Human Development*, *48*(4), 517-527. https://doi.org/10.1007/s10578-016-0679-7

Huang, K.-Y., Bornheimer, L. A., Dankyi, E., & de GraftAikins, A. (2018). Parental wellbeing, parenting and child development in Ghanaian families with young children. *Child Psychiatry and Human Development*, *49*, 833-841. https://doi.org/10.1007/s10578-018-0799-3

Jandric, S., & Kurtovic, A. (2021). Parenting sense of competence in parents of children with and without intellectual disability. *Europe's Journal of Psychology*, *17*(2), 75-91. https://doi.org/10.5964/ejop.3771

Jensen, E. W., James, S. A., Boyce, W. T., & Hartnett, S. A. (1983). The Family Routines Inventory: Development and validation. *Social Science and Medicine*, *17*, 201-211. https://doi.org/210.1016/0277-9536(1083)90117-X.

Johnson, B. E., & Ray, W. (2016). Family systems theory. In S. Smith (Ed.), *The Wiley Blackwell encyclopedia of family studies* (pp. 782-787). Wiley-Blackwell Publishing.

Jordan, S. S. (2003). *Further validation of the child routines inventory (CRI): Relationship to parenting practices, maternal distress and child externalizing behavior*. [Doctoral Dissertation, Louisiana State University]. https://digitalcommons.lsu.edu/gradschool_dissertations/3308/

Karlioglu, A., & Sari, H. Y. (2019). Zihinsel engelli cocuga sahip babalarda ebeveyn oz yeterliligi algianan sosyal destek ve aile yuku Ilisisi (The relationship between parental self-efficacy, perceived social support and family burden on fathers of children with intellectual disability). *Hemsirelikte Egitim Ve Dergisi (Journal of Education and Research in Nursing)*, *16*(2), 112-118. https://jer-nursing.org/Content/files/sayilar/75/KUHEAD_16_2_112_118.pdf

Kelly, S. J., Whitley, D. M., & Campos, P. E. (2011). Behavior problems in children raised by grandmothers: The role of caregiver distress, family resources, and the home environment. *Children and Youth Services Review*, *33*, 2138-2145. https://doi.org/10.1016/j.childyouth.2011.06.021

Kerig, P. K. (2019). Parenting and family systems. In M. H. Bornstein (Ed.), *Handbook of parenting: Being and becoming a parent* (3rd ed., Vol. 3, pp. 3-35). Routledge/Taylor & Francis Group.

Kilmer, R. P., Cook, J. R., Munsell, E. P., & Salvador, S. K. (2010). Factors associated with positive adjustment in siblings of children with severe emotional disturbance: The role of family resources and community life. *American Journal of Orthopsychiatry*, *80*, 473-481. https://doi.org/10.1111/j.1939-0025.2010.01050.x

Kim, J.-I. (2007). *Mothers' depression and parenting efficacy among economically disadvantaged Korean women: Test of a mediational model*. [Doctoral Dissertation, University of Pittsburgh]. http://d-scholarship.pitt.edu/8792/1/kimji071007rev.pdf

Kohl, G. O., Lengua, L. J., & McMahon, R. J. (2000). Parent involvement in school: Conceptualizing multiple dimensions and their relations with family and demographic risk factors. *Journal of School Psychology*, *38*(6), 501-523. https://doi.org/10.1016/S0022-4405(00)00050-9

Lee, C.-Y. S., Anderson, J. R., Horowitz, J. L., & August, G. J. (2009). Family income and parenting: The role of parental depression and social support. *Family Relations*, *58*, 417-430. https://doi.org/10.1111/j.1741-3729.2009.00563.x

Letiecq, B. L., & Koblinsky, S. A. (2003). African-American fathering of young children in violent neighborhoods: Paternal protective strategies and their predictors. *Fathering*, *1*, 215-237. https://doi.org/10.3149/fth.0103.215

Lindsey, R. A., & Barry, T. D. (2018). Protective factors against distress for caregivers of a child with autism spectrum disorder. *Journal of Autism and Developmental Disabilities*, *48*, 1092-1107. https://doi.org/10.1007/s10803-017-3372-1

Loor, M., & McNair, D. M. (1988). *Profile of Mood States Manual*. Educational and Industrial Testing Service.

Lovejoy, M. C., Weis, R., O'Hare, E., & Rubin, E. C. (1999). Development and initial validation of the Parent Behavior Inventory. *Psychological Assessment*, *11*(4), 534-545. https://doi.org/510.1037/1040-3590.1011.1034.1534.

Lovibond, S. H., & Lovibond, P. F. (1995). *Manual for the Depression Anxiety Stress Scales*. Australia Psychology Foundation.

Mahmoud, A. M., Osman, L. H., Gaafar, M. M. S., & Gomaa, N. I. H. (2022). Depression, burden, and self-efficacy among caregiving parents of children with cancer. *Egyptian Journal of Health Care*, *13*(2), 385-401. https://doi.org/10.21608/EJHC.2022.230387

McRae, E., Stoppelbein, L., O'Lelley, S., Fite, P., & Smith, S. (2020). Comorbid internalizing and externalizing symptoms among children with ADHD: The influence of parental distress, parenting practices, and child routines. *Child Psychiatry and Human Development*, *51*, 813-826. https://doi.org/10.1007/s10578-020-01019-z

Merson, E. S. (2012). *Mothers of children with ADHD: Does employment help?* [Doctoral Dissertation, University of Maryland]. https://drum.lib.umd.edu/bitstream/handle/1903/12994/Merson_umd_0117E_13282.pdf;jsessionid=1324CA19D39545EF12C5FF84FD987F4F?sequence=1

Mitchell, A. E. (2011). *The relationship between parents' self-efficacy belief, outcome expectations, child behaviour, and management of Atopic Dermatitis*. [Doctoral Dissertation, Queensland University of Technology]. https://eprints.qut.edu.au/47319/

Nievar, M. A., Jacobson, A., & Dier, S. (2008, November). Home visiting for at-risk preschoolers: A successful model for Latino families. Paper presented at the Annual Conference of the National Council on Family Relations, Little Rock, AR. https://files.eric.ed.gov/fulltext/ED502647.pdf.

Okado, Y., Bierman, K. L., & Welsh, J. A. (2014). Promoting school readiness in the context of socio-economic adversity: Associations with parental demoralization and support for learning. *Child Youth Care Forum*, *43*, 353-371. https://doi.org/10.1007/s10566-013-9242-x

Palermo, F., Ispa, J. M., Carlo, G., & Streit, C. (2017). Economic hardship during infancy and U.S. Latino preschoolers' sociobehavioral health and academic readiness. *Developmental Psychology*, *54*(5), 890-902. https://doi.org/10.1037/dev0000476

Piehler, T. F., Lee, S. S. W., Bloomquist, M. L., & August, G. A. (2014). Moderating effects of parental well-being on parenting efficacy outcomes by intervention delivery model of the early risers conduct problems prevention program. *Journal of Primary Prevention*, *35*(5), 321-337. https://doi.org/10.1007/s10935-014-0358-z

Platt, C., Roper, S. O., Mandleco, B., & Freeborn, D. (2014). Sibling cooperative and externalizing behaviors in families raising children with disabilities. *Nursing Research*, *63*(4), 235-242. https://doi.org/10.1097/NNR.0000000000000046

Podjarny, G. (2007). *Perceptions of parent-child relationship quality in parents of children with and without Autism*. [Master's Thesis, Carleton University]. https://curve.carleton.ca/system/files/etd/c55b0fd4-2883-47df-b920-66530c5be569/etd_pdf/d553b8e44edfee932cc420b8b45347c7/podjarny-perceptionsofparentchildrelationshipquality.pdf

Quinn, N. (2017). *Questioning mindful parenting as an intervention for parents of children diagnosed with Autism: Evidence for cognitive flexibility and resilience as precursory requirements*. [Honours Thesis, Australian College of Applied Psychology]. https://www.researchgate.net/publication/320854062_Questioning_mindful_parenting_as_an_intervention_for_parents_of_children_diagnosed_with_Autism_Evidence_for_cognitive_flexibility_and_resilience_as_precursory_requirements

Quittner, A. L., Steck, J. T., & Rouiller, R. I. (1991). Cochlear implants in children: A study of parental stress and adjustment. *American Journal of Otology*, *12*, 95-104. https://www.researchgate.net/profile/Alexandra-Quittner/publication/21087227_Cochlear_implants_in_children_A_study_of_parental_stress_and_adjustment/links/02e7e530e0bdc8e289000000/Cochlear-implants-in-children-A-study-of-parental-stress-and-adjustment.pdf

Radloff, L. S. (1977). The CES-D scale: A self-report depression scale for research in the general population. *Applied Psychological Measurement*, *1*, 385-401. https://doi.org/310.1177/014662167700100306

Raisanen, E. D. (2013). *The role of hardiness, family hardiness, and parenting self-efficacy on parenting stress in adoptive parents*. [Master's Thesis, University of Southern Mississippi]. https://aquila.usm.edu/masters_theses/518/

Richter, N., Bondu, R., Spiess, C. K., Wagner, G. G., & Trommsdorff, G. (2018). Relations among maternal life satisfaction, shared activities, and child well-being. *Frontiers in Psychology*, *9*, Article 739. https://doi.org/10.3389/fpsyg.2018.00739

Robinson, C. C., Mandleco, B., Olsen, S. F., & Hart, C. H. (2001). The Parenting Dimensions Questionnaire. In J. Touliatos, B. F. Perlmutter, & M. A. Straus (Eds.), *Handbook of family measurement techniques* (pp. 319-321). Sage Publications.

Robinson, S. M. (2019). *Understanding social support for parents of individuals with Autism Spectrum Disorders*. [Doctoral Dissertation, York University]. https://yorkspace.library.yorku.ca/xmlui/bitstream/handle/10315/36736/Robinson_Suzanne_M_2019_PhD.pdf?sequence=2&isAllowed=y

Robokos, D. (2012). *Cognitive, language, and social-emotional development among infants and toddlers in Early Head Start: An examination of the impact of cumulative risk*. KS Omniscriptum Publishing.

Rodenburg, R., Meijer, A. M., Dekovic, M., & Aldenkamp, A. P. (2007). Parents of children with enduring epilepsy: Predictors of parenting stress and parenting. *Epilepsy & Behavior*, *11*, 197-207. https://doi.org/10.1016/j.yebeh.2007.05.001

Rogers, M. A., Wiener, J., Marton, I., & Tannock, R. (2009). Supportive and controlling parental involvement as predictors of children's academic achievement: Relations to children's ADHD symptoms and parenting stress. *School Mental Health: A Multidisciplinary Research and Practice Journal*, *1*(2), 89-102. https://doi.org/10.1007/s12310-009-9010-0

Rudelli, N., Straccia, C., & Petitpierre, G. (2021). Fathers of children with autism spectrum disorder: Their perceptions of paternal role as a predictor of caregiving satisfaction, self-efficacy and burden. *Research in Autism Spectrum Disorders*, *83*, Article 101744. https://doi.org/10.1016/j.rasd.2021.101744

Sajedi, F., Ahmadi Doulabi, M., Vameghi, R., Akbarzadeh Baghban, A., Rashidi Fakari, F., & Mazaheri, M. A. (2020). Social determinants of health, maternal involvement, and child development: Direct and mediated pathways. *Iranian Journal of Child Neurology*, *14*(4), 63-76. https://www.ncbi.nlm.nih.gov/pmc/articles/PMC7660026/pdf/ijcn-14-063.pdf

Santiago, R. T. (2019). *Examining parent-teacher relationship quality and family involvement for children with Autism Spectrum Disorder*. [Doctoral Dissertation, University of Oregon]. https://scholarsbank.uoregon.edu/xmlui/bitstream/handle/1794/24816/Santiago_oregon_0171A_12159.pdf?sequence=1

Scheier, M. F., Carver, C. S., & Bridges, M. W. (1994). Distinguishing optimism from neuroticism (and trait anxiety, self-mastery, and self-esteem): A reevaluation of the Life Orientation Test. *Journal of Personality and Social Psychology*, *67*(6), 1063-1078. https://doi.org/10.1037/0022-3514.67.6.1063

Semke, C. A., Garbacz, S. A., Kwon, K., Sheridan, S. M., & Woods, K. E. (2010). Family involvement for children with disruptive behaviors: The role of parenting stress and motivational beliefs. *Journal of School Psychology*, *48*, 293-312. https://doi.org/10.1016/j.jsp.2010.04.001

Seymour, M., Giallo, R., Cooklin, A., & Dunning, M. (2015). Maternal anzxety, risk factors and parenting in the first post-natal year. *Child: Care, Health and Development*, *41*(2), 314-323. https://doi.org/10.1111/cch.12178

Shackell, E. (2011). *Spirituality and religion as coping mechanisms: Families raising children with developmental disabilities*. [Master's Thesis, Saint Paul University]. https://ruor.uottawa.ca/bitstream/10393/20007/1/Shackell_Erin_2011_thesis.pdf

Shamash, E. R. (2011). *Predictors of stress among parents of preschool aged children with Autism Spectrum Disorders*. [Doctoral Dissertation, Columbia University]. https://www.proquest.com/docview/903268838/fulltextPDF/C23515A17B2D4CD6PQ/1?accountid=8385

Shelton, K. K., Frick, P. J., & Wootton, J. (1996). Assessment of parenting practices in families of elementary school-age children. *Journal of clinical child psychology*, *25*(3), 317-329. https://doi.org/10.1207/s15374424jccp2503_8

Sheras, P. L., Abidin, R. R., & Konold, T. R. (1998). *Stress Index for Parents of Adolescents: Professional manual*.

Shine, R. (2014). *Parent and family factors related to children's progress in intensive behavioural intervention*. [Master's Thesis, York University]. https://yorkspace.library.yorku.ca/xmlui/bitstream/handle/10315/28195/Shine_Rebecca_N_2014_Masters.pdf?isAllowed=y&sequence=2

Shoshani, A., & Yaari, S. (2022). Parental flow and positive emotions: Optimal experiences in parent-child interactions and parents' well-being. *Journal of Happiness Studies*, *23*, 789-811. https://doi.org/10.1007/s10902-021-00427-9

Sosinsky, L., Marakovitz, S., & Carter, A. (2004). Parent–Child Interaction Rating Scales (PCIRS). *Unpublished Manual, University of Massachusetts Boston*.

Stein, R. E. K., & Jessop, D. J. (2003). The Impact on Family Scale revisited: Further psychometric data. *Developmental and Behavioral Pediatrics*, *24*, 9-16. https://doi.org/10.1097/00004703-200302000-00004

Suurmond, R., van Rhee, H., & Hak, T. (2017). Introduction, comparison, and validation of *Meta-Essentials*: A free and simple tool for meta-analysis. *Research Synthesis Methods*, *8*, 537-553. https://doi.org/10.1002/jrsm.1260

Sytsma, S. E., Kelley, M. L., & Wymer, J. H. (2001). Development and validation of the Child Routines Inventory. *Journal of Psychopathology and Behavioral Assessment*, *23*, 241-251. https://doi.org/10.1023/A:1012727419873

Taraban, L., Shaw, D. S., Leve, L. D., Wilson, M. N., Dishion, T. J., Natsuaki, M. N., & Neiderhiser, J. M. (2017). Maternal depression and parenting in early childhood: Contextual influence of marital quality and social support in two samples. *Developmental Psychology*, *53*(3), 436-449. https://doi.org/10.1037/dev0000261

Taylor, L. J., Luk, S. Y. L., Leadbetter, K., Moore, H. L., & Charman, T. (2021). Are child autism symptoms, developmental level and adaptive function associated with caregiver feelings of wellbeing and efficacy in the parenting role? *Research in Autism Spectrum Disorders*, *83*, Article 101738. https://doi.org/10.1016/j.rasd.2021.101838

Taylor, R. D., Lopez, E. I., Budescu, M., & McGill, R. K. (2012). Parenting practices and adolescent internalizing and externalizing problems: Moderating effects of socially demanding kin relations. *Journal of Child and Family Studies*, *21*(3), 474-485. https://doi.org/10.1007/s10826-011-9501-0

Taylor, Z. E., Widaman, K. F., Robins, R. W., Jochem, R., Early, D. R., & Conger, R. D. (2012). Dispositional optimism: A psychological resource for Mexican-origin mothers experiencing economic stress. *Journal of Family Psychology*, *26*(1), 133-139. https://doi.org/10.1037/a0026755

Tichovolsky, M. H., Arnold, D. H., & Baker, C. N. (2013). Parent predictors of changes in child behavior problems. *Journal of Applied Developmental Psychology*, *34*(6), 336-345. https://doi.org/10.1016/j.appdev.2013.09.001

Tobing, L. E. (2004). *Stress, coping, and psychological distress of mothers of children with pervasive developmental disorders*. [Doctoral Dissertation, Fordham University]. https://research.library.fordham.edu/dissertations/AAI3159399/

Trivette, C. M., & Dunst, C. J. (1992). Characteristics and influences of role division and social support among mothers of preschool children with disabilities. *Topics in Early Childhood Special Education*, *12*(3), 367-385. https://doi.org/10.1177/027112149201200308

Trivette, C. M., Dunst, C. J., & Hamby, D. W. (2010). Influences of family-systems intervention practices on parent-child interactions and child development. *Topics in Early Childhood Special Education*, *30*, 3-19. https://doi.org/10.1177/0271121410364250

Van Rhee, H. J., Suurmond, R., & Hak, T. (2015). *User manual for Meta-Essentials: Workbooks for meta-analysis (Version 1.4)*. Erasmus Research Institute of Management, The Netherlands. www.erim.eur.nl/research-support/meta-essentials

Viechtbauer, W., & Cheung, M. W.-L. (2010). Outlier and influence diagnostics for meta-analysis. *Research Synthesis Methods*, *1*, 112-1125. https://doi.org/10.1002/jrsm.11

Wade, S. L., Taylor, H. G., Walz, N. C., Salisbury, S., Stancin, T., Bernard, L. A., . . . Yeates, K. O. (2008). Parent-child interactions during the initial weeks following brain injury in young children. *Rehabilitation Psychology*, *53*(1), 180-190. https://doi.org/10.1037/0090-5550.53.2.180

Weiss, J. A., & Lunsky, Y. (2011). The Brief Family Distress Scale: A measure of crisis in caregivers of individuals with autism spectrum disorders. *Journal of Child and Family Studies*, *20*(4), 521-528. https://doi.org/10.1007/s10826-010-9419-y

Whittaker, J. E. V., Harden, B. J., See, H. M., Meisch, A. D., & Westbrook, T. R. (2011). Family risks and protective factors: Pathways to Early Head Start toddlers' social-emotional functioning. *Early Childhood Research Quarterly*, *26*, 74-86. https://doi.org/10.1016/j.ecresq.2010.04.007

Winstone, L. K., Curci, S. G., & Crnic, K. A. (2021). Pathways to maternal and child well-being: Stability and transaction across toddlerhood. *Parenting: Science and Practice*, *21*(2), 118-140. https://doi.org/10.1080/15295192.2019.1701933

Wood, Z. B. (2012). *Public child welfare adoption: Adolescent adoptee perception of adoption outcomes*. [Doctoral Dissertation, Case Western Reserve University]. http://rave.ohiolink.edu/etdc/view?acc_num=case1340289839

Woods, K. (2011). *Examining the effect of medical risk, parental stress, and self-efficacy on parent behaviors and the home environment of premature children*. [Doctoral Dissertation, University of Nebraska]. http://digitalcommons.unl.edu/cehsdiss/130

World Health Organization. (1996). *WHOQOL-BREF: Introduction, administration, scoring and generic version of the assessment*. World Health Organization. https://www.who.int/mental_health/media/en/76.pdf

Yeung, J. W. K., & Chan, Y.-C. (2010). The mediating role of parental psychological health in Chinese families in an impoverished context in Hong Kong. *Journal of General Social Issues*, 1085-1100. https://doi.org/10.5559/di.20.4.08

Yurdusen, S., Erol, N., & Gencoz, T. (2013). The effects of parental attitudes and mothers' psychological well-being on the emotional and behavioral problems of their preschool children. *Maternal and Child Health Journal*, *17*, 68-75. https://doi.org/10.1007/s10995-012-0946-6
